# The promise of γδ T cells and the γδ T cell receptor for cancer immunotherapy

**DOI:** 10.1038/cmi.2015.28

**Published:** 2015-04-13

**Authors:** Mateusz Legut, David K Cole, Andrew K Sewell

**Affiliations:** Division of Infection and Immunity and Systems Immunity University Research Institute, Cardiff University School of Medicine, Cardiff, UK

**Keywords:** cancer, γδ T cells, immunotherapy, phosphoantigens, T cell receptor

## Abstract

γδ T cells form an important part of adaptive immune responses against infections and malignant transformation. The molecular targets of human γδ T cell receptors (TCRs) remain largely unknown, but recent studies have confirmed the recognition of phosphorylated prenyl metabolites, lipids in complex with CD1 molecules and markers of cellular stress. All of these molecules are upregulated on various cancer types, highlighting the potential importance of the γδ T cell compartment in cancer immunosurveillance and paving the way for the use of γδ TCRs in cancer therapy. Ligand recognition by the γδ TCR often requires accessory/co-stimulatory stress molecules on both T cells and target cells; this cellular stress context therefore provides a failsafe against harmful self-reactivity. Unlike αβ T cells, γδ T cells recognise their targets irrespective of HLA haplotype and therefore offer exciting possibilities for off-the-shelf, pan-population cancer immunotherapies. Here, we present a review of known ligands of human γδ T cells and discuss the promise of harnessing these cells for cancer treatment.

## INTRODUCTION

The adaptive immune compartment comprises of three distinct cell subsets, namely B cells, T cells expressing an αβ T cell receptor (TCR) and T cells expressing a γδ TCR. These cells generate their defining B cell receptors (BCRs), or TCRs using somatic V(D)J recombination which enables them to recognise a vast spectrum of antigens. Despite the fact that gene segments used in rearrangement leading to αβ and γδ TCRs were discovered almost at the same time,^1^ the elucidation of αβ T cell biology progressed rapidly, while the complexities of γδ T cells have been slower to emerge. Nevertheless, the tripartite organisation of the lymphocytic immune system appears to be a fundamental requirement for efficient function, as this organisation is present both in jawed and jawless vertebrates, even though the manner of generating the defining receptors completely differs between these lineages.^2^ The unique functions of γδ T cells, particularly in terms of antigen recognition and kinetics of response, provide further evidence that γδ T cells represent an important and non-redundant lymphocyte subset.

**Figure 1:**
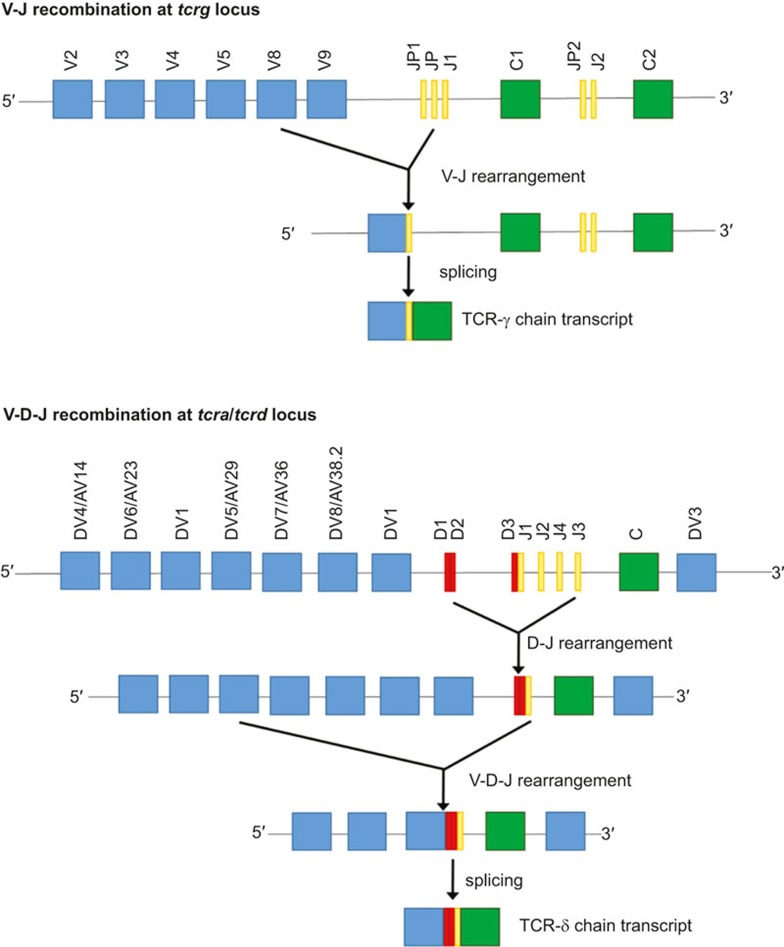
V(D)J recombination at the *tcrg* (upper panel) and *tcra/tcrd* (lower panel) locus. Only the functional gene segments are shown. The TCR-γ chain is produced using only a single V-J recombination, with P/N additions occurring at the V-J junction. The TCR-δ chain is produced using V-D-J recombinations that can involve either 2 or 3 D segments, leading to the creation of up to 4 N diversity regions. For the clarity of the figure, only the gene segments that can be used in TCR-δ chain production are presented (lower panel). The organisation of loci *tcrg* and *tcra/tcrd* was adapted from IMGT database.^3^

**Figure 2:**
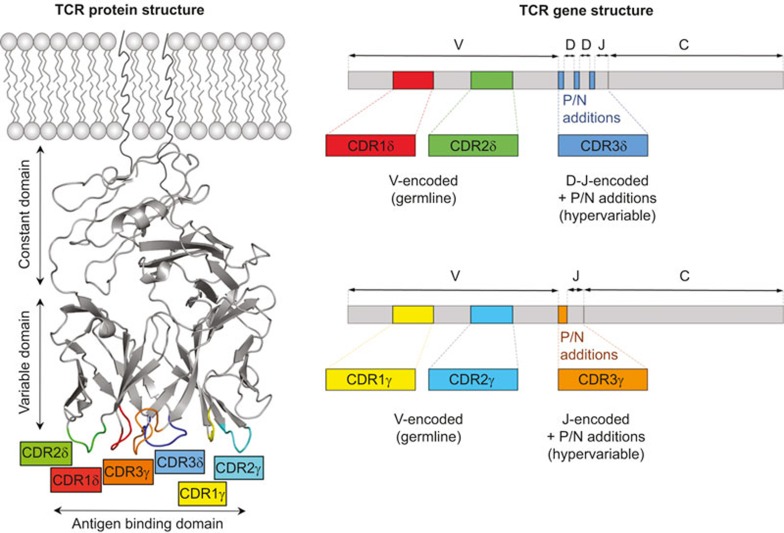
9C2 γδ TCR protein structure (left panel) and γ and δ chain mRNA architecture (right panel). The CDR loops are colour-coded. PDB ID: 4LHU.^37^

V(D)J recombination of the γ and δ chain genes is shown in Figure 1 (IMGT nomenclature^3^). There are 4–6 (depending on haplotype) functional variable (V)γ, and 8 Vδ gene segments in humans.^4^ Some Vδ segments can be used to generate both the α and δ chains of the TCR, as they are located within the *tcra* locus.^5,6^ The number of V segments that can be used for γδ T cells is much smaller than that for αβ T cells (46 Vα and 48 Vβ segments). However, the potential diversity of γδ TCR surpasses that of αβ TCR, owing to extensive N-region nucleotide additions and presence of distinct D segments (present only in *tcrd* but not *tcrg* locus) which can be used simultaneously and read in all three frames. This junctional variability results in the generation of hyperdiversity focused on the complementarity determining region (CDR)3 loops which are crucial for antigen recognition.^7^ Furthermore, the length of the CDR3s of both α and β chains is constrained, due to the requirement to make a well-defined contact with peptide-MHC complexes, while CDR3 in the δ chain is usually more variable and longer than its γ counterpart.^8^ With regard to CDR3 length, the γδ TCR resembles the BCR more than αβ TCR. This greater variability of γδ TCRs may translate into recognition of both proteins and smaller molecules. The CDRs form loops in the γδ TCR structure to provide a highly variable antigen-binding domain at the membrane-distal end of the molecule (Figure 2).

After arising from a common progenitor in the thymus, the maturation pathways of γδ and αβ T cells diverge. Notably, the development of γδ TCR^+^ thymocytes does not require the expression of Aire,^9^ a transcriptional regulator crucial for the negative selection of autoreactive αβ T cells. The mechanism by which T cells become committed to the αβ or γδ lineage is not yet fully understood as thymocytes rearrange β, γ and δ genes at the same time which can lead to simultaneous expression of the γδ TCR and pre-TCR (invariant Tα paired with TCR-β).^10^ However, recent evidence suggests that thymocytes adopt the γδ T cell lineage after receiving a strong signal via γδ TCR, which can be additively enforced by additional signalling via pre-TCR – thus enabling weak ligands to drive γδ T cell lineage commitment as well.^11^ If cells fail to receive this survival signal they silence the γδ TCR and undergo TCR-α rearrangement.^12^ This signal strength model implies that γδ T cells need to encounter a cognate ligand in the thymus. However, to date only one molecule, namely Skint-1, has been described as a thymically expressed ligand necessary for development of a subset of mouse γδ T cells.^13^ The identity of other ligands required for positive selection of γδ remains to be elucidated. Strong γδ TCR-mediated interactions in the thymus have been shown to result in upregulation of CD73, the earliest identified marker of γδ lineage commitment.^14^ CD73 is expressed by the vast majority of γδ T cells in the periphery, supporting the notion that recognition of the ligand in the thymus is a common occurrence in γδ T cell development. Another striking difference in development between αβ and γδ T cells is the acquisition of effector functions. Conventional αβ T cells acquire their effector phenotype, in terms of produced cytokines, upon interactions with their targets in the periphery, while γδ T cell functions, like their anatomical location, appear to be pre-determined in the thymus by the chain usage of their TCR.^15^

In humans, γδ T cells constitute 0.5–10% of T cells in peripheral blood but are substantially enriched in epithelial tissues (e.g. in skin, lungs, intestine). The majority of peripheral blood γδ T cells express the Vδ2 chain, while the tissue-resident γδ T cells are mainly Vδ1^pos^ or Vδ3^pos^. The precise reasons for this tissue specificity and the mechanisms that underlie it are yet to be elucidated. The role of γδ T cells in infection is well established and has been amply reviewed elsewhere.^16^ More recent discoveries suggest that γδ T cells play a role in anticancer immunity, and it has been known for over a decade that mice lacking the γδ TCR are more susceptible to some cancers through unknown mechanisms.^17^ Here, we review recent advances in discovering cancer-associated antigens recognised by human γδ T cells and the evidence that there is a broad spectrum of γδ T cell ligands waiting to be discovered. We also provide a brief overview of therapeutic applications of γδ T cells in cancer immunotherapy.

## γδ TCR RECOGNITION OF CANCER-ASSOCIATED ANTIGENS

Known ligands of human γδ TCR are scarce when compared with the vast spectrum of antigens recognised by αβ T cells. However, significant progress in this area has been made in the recent years, linking the observed recognition of tumour cells to specific ligands confirmed by biochemical and biophysical data. The main targets encompass phosphorylated prenyl antigens, endo- and exogenous lipids presented by CD1-family proteins, and cell stress molecules that can indicate DNA damage, viral infection or malignant transformation.

### Recognition of phosphoantigens

Phosphorylated isoprenoid metabolites, commonly referred to as phosphoantigens (PAgs), can be produced by both bacterial and eukaryotic cells, using non-mevalonate and mevalonate biosynthetic pathways, respectively. The accumulation of PAgs, such as isopentenyl pyrophosphate (IPP), is a result of metabolic dysregulation that commonly occurs in tumour cells^18^ and therefore the enzymes of the mevalonate pathway are a valid target for anticancer drugs (reviewed in Clendening and Penn^19^). Importantly, PAgs have long been known to be recognised by γδ T cells expressing Vγ9Vδ2 TCR. The presence of this peripheral blood subset of γδ T cells is restricted to higher primates, with a few exceptions.^20^ Importantly, mice and other rodents do not possess any corresponding T cell subsets that respond to PAgs.

**Figure 3:**
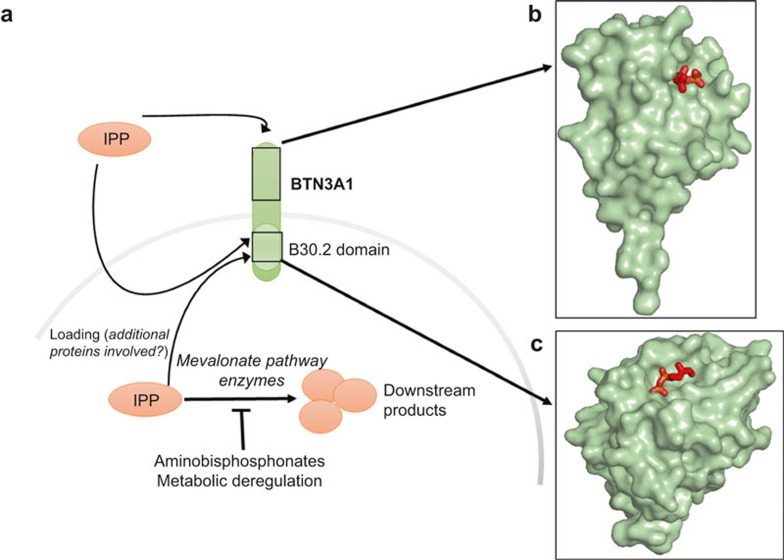
(**a**) Schematic representation of the phosphoantigen presentation pathways by human cells showing phosphoantigen (IPP) binding in the extracellular pocket of BTN3A1 (**b**) and phosphoantigen (cHDMAPP) binding in the intracellular domain (B30.2) of BTN3A1 (C). PDB IDs: 4JKW (B)^31^ and 4N7U (C).^29^

The mode of PAg recognition has only recently been resolved. Early studies indicated that recognition of PAgs by human Vγ9Vδ2 T cells required cell surface presentation by a species-specific molecule.^21^ One of the proposed molecules presenting the antigens was F1-ATPase which is expressed on the surface of a wide range of tumour cells.^22^ Further research suggested that PAgs could bind F1-ATPase in the form of a nucleotide derivative, increasing the efficiency of T cell activation. However, as antibody blocking of F1-ATPase did not abrogate T cell recognition, other molecules were thought to be involved in PAg presentation.^23^ The cell surface-expressed Butyrophilin molecules are encoded within the MHC class I locus and offer attractive potential candidates as PAg-presenting molecules. Butyrophilin-3A (BTN3A/CD277) is present in humans in three isoforms (BTN3A1, BTN3A2 and BTN3A3).^24^ Recent studies have shown that antibodies specific for BTN3A1 could either mimic PAg-mediated activation of the TCR (antibody 20.1) or abrogate this stimulatory effect (antibody 103.2).^25^ Biophysical analysis of the underlying interactions suggested that 20.1 antibody induced/stabilised a TCR-activating conformation of BTN3A1.^26^ Additionally, PAgs and 20.1 antibody activated the same intracellular signalling pathways in the responding γδ T cells, suggesting a common recognition process.^27^ Interestingly, transduction studies revealed that while the expression of BTN3A1 alone is sufficient for the activation mediated by 20.1, additional genes located on chromosome 6 are required for PAg-mediated recognition.^28^ These studies suggest the possible existence of molecules responsible for efficient loading of PAgs into butyrophilin-3A1 in a conceptually similar manner to loading peptides into MHC, or that there is a requirement for a co-stimulatory ligand. Two main models explaining the role of BTN3A1 have been proposed, involving intracellular or extracellular binding of pyrophosphate antigens (Figure 3a). Sandstrom *et al.* demonstrated that BTN3A1 acts as a sensor of intracellular PAgs by binding them in a surface pocket located in an intracellular domain termed B30.2.^29^ This result was confirmed by the crystal structure of the B30.2-PAg complex (Figure 3c) and by mutational analysis. Introduction of the B30.2 domain of BTN3A1 into the non-activating isoform BTN3A3 was shown to confer PAg binding and T cell stimulation. This result falls in line with observations by Wang *et al.* who demonstrated a lack of high-affinity binding of PAgs to the extracellular regions of BTN3A1.^30^ In direct contrast, De Libero and colleagues resolved the crystal structure of two PAgs (IPP and HMBPP) in complex with the extracellular domain of BTN3A1 where the antigen was bound in a shallow surface pocket (Figure 3b).^31^ The binding affinity of Vγ9Vδ2 TCR to BTN3A1 was relatively weak (K_D_ < 0.1 mM) but increased in the presence of IPP. These two mechanisms need not be mutually exclusive and it remains possible that PAgs bind to both the intracellular and extracellular domains of BTN3A1 to provide a failsafe against aberrant TCR triggering and autoimmunity. The binding affinities of IPP to BTN3A1 and the Vγ9Vδ2 TCR interaction with BTN3A1-IPP are summarised in Table 1. Further investigation will be required to determine the exact mechanism of antigen presentation by BTN3A1.

### γδ T cells recognise specific ligands in the context of antigen-presenting molecules from the CD1 family

**Figure 4:**
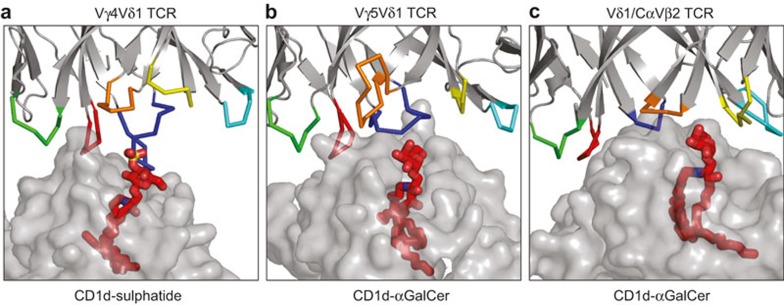
Complex formation between TCR and CD1 ligands. CDR loops are colour coded as in Figure 2: CDR1δ-red, CDR2δ-green, CDR3δ-blue, CDR1γ-yellow, CDR2γ-cyan and CDR3γ-orange. The blue loop in panel C is CDR3δ/α. PDB IDs: 4MNG (A),^36^ 4LHU (B),^37^ 4WO4 (C).^38^

Four members of the human CD1 family of proteins (CD1a–CD1d) present both endogenous and exogenous lipids that can be recognised by both αβ and γδ T cells.^32^ Early reports demonstrated that mucosal γδ T cell clones showed broad reactivity towards these four CD1 isoforms loaded with exogenous phospholipids.^33^ Further studies showed that T cells expressing the Vδ3 chain were capable of killing tumour cells expressing CD1d (but not other CD1 isoforms) without addition of exogenous lipids.^34^ Additionally, the γδ T cell compartment of peripheral blood was shown to recognise CD1d in complex with self-lipids termed sulphatides (sulphated galactosylceramides).^35^ CD1d-reactive γδ T cells expressed similar Vδ1-Jδ1 chains paired with different Vγ chains. Notably, some of the generated clones could bind CD1d without any loaded lipid. Recent studies provided the structural basis for CD1d-sulphatide recognition, demonstrating that the binding occurred solely via the δ chain of the TCR – the germline-encoded residues made contact with the CD1d molecule whereas the CDR3δ loop interacted with sulphatide (Figure 4a).^36^ As the CDR3 region is the most variable part of the γδ TCR, it is possible that a subset of γδ T cells can recognise diverse lipid cargoes presented by CD1 molecules, in a similar way to αβ TCR recognition of peptides bound to MHC. A similar mode of interaction was reported for CD1d-α-galactosylceramide (αGalCer) and a Vδ1 TCR (9C2) where CD1d was bound by germline-encoded residues in the δ chain while the lipid was recognised by the hypervariable CDR3γ loop (Figure 4b).^37^ Interestingly, Pellicci *et al.* have recently discovered a novel T cell subgroup recognising CD1d-αGalCer complex, expressing a Vδ1 domain linked to permissive Jα-Cα segments, paired with diverse TCR-β chains (termed δ/αβ T cells).^38^ Again, the binding of the TCR to CD1d was mainly driven by germline-encoded residues in the Vδ1 domain while the specific recognition of the bound lipid was exclusively mediated by the β chain of the TCR (Figure 4c). The affinity of the interaction between TCRδ/αβ and CD1d-αGalCer was, however, much higher than described for 9C2 γδ TCR (the binding affinities of TCRs to CD1d complexed with lipid ligands are summarised in Table 2). Importantly, functional δ/αβ T cells comprise a high proportion of human CD1d-reactive T cells. αGalCer has been so far investigated only in context of boosting NK T cell anticancer activity,^39^ but it could potentially be used to activate αGalCer-reactive γδ T cells, similarly to using PAgs. Notably, a recent study showed that small quantities of αGalCer and other α-glycosylceramides can be endogenously produced in mammalian species to modulate the immune response.^40^ Additionally, CD1d may be an important target for cancer immunotherapy as its high expression was reported on chronic lymphocytic leukaemia cells, correlating with disease progression.^41^ Similarly, CD1c on acute leukaemia cells was shown to present a novel class of immunogenic self-lipids, called methyl-lysophosphatidic acids (mLPAs). Recognition of CD1c-mLPA complex by γδ T cells has yet to be demonstrated,^42^ but recent studies showing that CD1 family members can present self-lipids make these molecules attractive targets for future exploration.

### γδ TCR recognition of general stress ligands

γδ T cells are known to expand in response to some viral infections, with cytomegalovirus (CMV) providing the best-studied example to date. CMV infection results in preferential expansion of γδ T cells expressing Vδ1, Vδ3 and Vδ5 (commonly termed ‘non-Vδ2 T cells').^43,44^ These cells can also recognise and lyse intestinal tumour cells, in line with the fact that they are enriched in epithelial tissues where they perform stress surveillance.^44^ Interestingly, infection with CMV, regarded as a treatment-related complication in kidney transplant recipients, serves a protective role against cancer in those patients.^45^ Taken together with the fact that Vδ2^neg^ compartment is selectively expanded in CMV carriers and does not diminish with age, it is possible that CMV-mediated activation of γδ T cells confers an adaptive-like, lifelong reduction of cancer risk.^46^

The ligands of dual reactive γδ T cell clones recognising CMV-infected and transformed cells remain unknown, with one notable exception. Recently, Déchanet-Merville and colleagues identified the ligand of one of such clone, named LES. The LES γδ T cell clone expressed Vγ4 and Vδ5 TCR chains and was substantially enriched (25% of peripheral blood T cells) in a CMV-infected transplant recipient. LES was shown to recognise a range of solid tumour lines and CMV-infected cells via its TCR rather than natural killer (NK)-type receptors. The LES TCR was demonstrated to bind endothelial protein C receptor (EPCR).^47^ EPCR exhibits sequence and structural homology with the MHC-like protein family CD1, and can present phospholipids bound in the antigen-presenting groove.^48^ EPCR plays a dual role in cancer, as it can both promote and inhibit metastases, presumably depending on whether it is expressed on tumour cells or endothelium (reviewed in Mohan Rao *et al.*^49^). Additionally, EPCR signalling can activate antiapoptotic or proapoptotic pathways through unresolved mechanisms. Importantly, direct binding of the LES TCR to EPCR was confirmed by surface plasmon resonance, showing relatively low binding affinity (K_D_ ≈ 90 µM) (Table 3). EPCR, however, does not appear to be a common, innate-like target for dual reactive γδ T cell clones, as the recognition of the tumour lines by such clones, isolated from other patients, was not abrogated by antibody blocking of EPCR. Moreover, the LES TCR recognition was mediated by the hypervariable CDR3 loop of the Vγ4 chain, supporting the hypothesis that EPCR binding was part of an adaptive response. Interestingly, the expression of EPCR was not sufficient to induce T cell activation, suggesting that functional recognition required a cell stress context and additional co-stimulatory molecules. Indeed, CMV infection conferred the recognition of fibroblasts and endothelial cells without altering EPCR expression suggesting that recognition of this receptor occurs in a stress-related co-stimulatory context. The identity of these co-stimulatory ligands remains unknown.

EPCR is not the only cell stress marker that requires an additional co-stimulatory context for efficient recognition by γδ T cells. Other examples described so far encompass the human homologue of the bacterial MutS (hMSH2) protein,^50^ being a part of the DNA mismatch repair system (MMR), and MHC class I polypeptide-related sequence A (MIC A),^51^ a broadly recognised stress marker. Both molecules are recognised by both highly variable γδ TCR and the invariant NKG2D receptor (discussed in the next section). hMSH2 is a key protein involved in repairing mutations resulting from DNA recombination or replication. Unsurprisingly, defects in hMSH2 have been reported in various tumour histologies including lung, colon, breast and prostate cancers.^52^ Although hMSH2 is normally restricted to the nucleus, it can be translocated during cellular stress.^53^ Recent studies have demonstrated that hMSH2 can be a ligand for some Vδ2 γδ T cells when it is ectopically expressed, inducing cytotoxicity and IFN-γ production.^54^ Surface expression of hMSH2 can be induced by Epstein-Barr virus (EBV) infection,^54^ oxidative stress and proinflammatory cytokines such as IL-18 corroborating its role in generalised stress.

Interestingly, oxidative stress promoted the expression of MIC A in a similar manner to hMSH2, involving p38/JNK signalling pathways.^55^ Another group reported MIC A/B upregulation resulting from ataxia telangiectasia mutated (ATM) protein kinase signalling in response to DNA damage, contributing to γδ T cell-mediated lysis of ovarian cancer cell lines.^56^ Additionally, the level of MIC A expression on breast cancer cell lines correlated with their susceptibility to γδ T cell-mediated cytotoxicity.^57^ Induction of surface expression of MIC A/B was also reported in primary glioblastoma after chemotherapy.^58^ EBV infection induces MIC A expression as well, showing further similarities between expression of MIC and hMSH2.

## γδ TCRs RECOGNISE TUMOURS IN A CO-STIMULATORY STRESS CONTEXT

**Figure 5:**
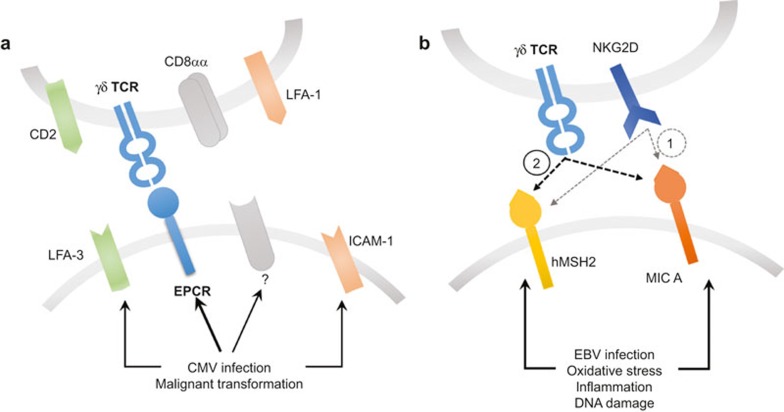
Recognition of γδ TCR ligands requires a cell stress context. (**a**) Recognition of EPCR requires co-stimulatory ligands on the surface of the target cells, and accessory molecules on the surface of the T cell. (**b**) Both hMSH2 and MIC A become upregulated and ectopically expressed in response to cell stress stimuli and are dually recognised by NKG2D and TCR. The sequential model of recognition implies that the initial contact provided by a transient high-affinity NKG2D–ligand interaction (1) is followed by formation of a stable low affinity TCR-ligand complex (2).

As mentioned above, the recognition of cell stress markers by γδ T cells appears not to be driven solely by the interaction between the TCR and its cognate ligand but rather requires a wider stress context provided by co-stimulatory receptors on the T cell and additional self-antigens. In the case of the LES clone recognising EPCR, part of the co-stimulatory effect was provided by LFA-1 on the T cells binding to ICAM-1 (overexpressed as a result of CMV infection).^47^ CD2 interaction with CD58 (LFA-3) was also implicated. Blocking of TCR binding to EPCR completely abrogated the recognition while disrupting the ICAM-1-LFA-1 and CD2-CD58 axes merely decreased T cell activation, demonstrating the dominance of TCR signalling and the possible co-existence of complementary co-stimulatory pathways. Furthermore, another group showed that the CD8αα homodimer could act as a coreceptor for recognition of CMV-infected cells by Vδ1 TCR chains (Figure 5a).^59^ This observation is in line with the findings that T cells expressing CD8αα play an important role in immunosurveillance of the epithelial tissues against viral^60^ and bacterial^61^ infections.

The requirement for co-stimulation is well established for αβ T cells and it has been known for some time that CD8αβ heterodimer acts to co-receive peptide-MHC class I, stabilising the TCR interaction^62^ and ensuring full phosphorylation of the CD3ζ chain^63^ to increase sensitivity to antigen by up to a million-fold^64^ (reviewed in Cole *et al.*^65^). Thus the αβ T cell co-receptors, CD4 and CD8, ensure that this T cell subset is MHC-restricted^66,67^ and determine the fate of the developing T cell.^68^ In an analogous fashion, co-stimulation via stress-induced ligands might maintain the correct focus and function of the γδ T cell compartment. In keeping with this concept, both hMSH2 and MIC A/B are dually recognised by cognate TCRs and the NKG2D receptor (Figure 5b). NKG2D is commonly expressed on the surface of NK cells, αβ T cells and γδ T cells, and binds to cell stress molecules from the UL16-binding protein (ULBP1–6) family (reviewed in Ullrich *et al.*^69^). The interactions of germline-encoded NKG2D with such diverse ligands are possible because of conserved fragments within the α1α2 superdomain in the ligands. The involvement of the γδ TCR and NKG2D in recognition of the MIC A molecule has recently been studied. Xu *et al.* showed that both TCR and NKG2D bound overlapping fragments of MIC A, with different affinity and kinetics – for NKG2D, the affinity was much higher than for the TCR (Table 3).^70^ Nevertheless, the complex between TCR and MIC A was particularly stable, suggesting a sequential model of binding where the initial contact by NKG2D is followed by the more stable TCR:MIC A complex. The role of TCR chains in complex formation remains to be fully resolved as the studies implicated partial contributions of the germline-encoded CDR loops from Vδ1 TCR^70^ while other studies showed that MIC A-associated recognition of tumour cells could be mediated by both Vδ1^71^ and Vδ2^56,57,72^ TCRs. Notably, TCR engagement has been shown to be indispensable for γδ T cell-mediated cytotoxicity whereas NKG2D played only a co-stimulatory role; thus indicating the importance of TCR:MIC A complex formation.^73^ ULBP molecules may be recognised in a similar manner as it has been shown that ULBP-4 engages both NKG2D and Vγ9Vδ2 TCRs.^74^

The sequential recognition of different targets by γδ T cells could therefore play an important role in immunosurveillance as it allows the T cell to rapidly scan the target cells for the signs of stress, indicating a possible infection or transformation. The requirement for a multicomponent stress context for full T cell activation could then provide fail-safe protection against autoimmunity. The apparent co-existence of diverse co-stimulatory axes decreases the chances of immune evasion.

## HARNESSING γδ T CELLS FOR THERAPY

### Stimulating γδ T cells *in vivo* with phosphoantigens

As mentioned above, the Vγ9Vδ2 T cell subset that predominates in human peripheral blood is capable of responding to prenyl pyrophosphates. This feature has been employed to redirect Vγ9Vδ2 T cells to tumours by manipulating isoprenoid metabolism in the cancer cells. Such manipulation can be achieved using aminobisphosphonates (e.g. zoledronate, pamidronate, risedronate) which are structural analogues of prenyl pyrophosphates that contain an amino moiety. As a result, aminobisphosphonates act indirectly by inhibiting farnesyl pyrophosphate synthase (FPPS) in the mevalonate pathway leading to the accumulation of prenyl pyrophosphate substrates.^75,76^ Recently, Idrees *et al*. screened over 50 tumour cell lines, showing a direct correlation between zoledronate-induced FPPS inhibition and tumour recognition by Vγ9Vδ2 T cells.^77^ The FPPS-inhibiting concentration of zoledronate was on average two orders of magnitude lower than the concentration required for inhibition of tumour proliferation, thus indicating that T cell activation was not the result of cell death. Another group showed that risedronate caused PAg accumulation in the tumour cells which were then recognised by Vγ9Vδ2 T cells.^78^ Upon recognition, T cells produced IFNγ which caused upregulation of ICAM-1 on tumour cells, contributing to a positive feedback loop and subsequent cytotoxicity. Additionally, aminobisphosphonates,^79^ as well as conventional chemotherapy, can sensitise the neoplastic cells to cytototoxic activity of Vγ9Vδ2 T cells. This may be an attractive therapeutic option for eradication of cancer-initiating cells, which are often resistant to conventional therapy and can contribute to the relapse of the disease. Finally, γδ T cells are known to acquire the phenotype of professional antigen-presenting cells upon activation.^80^ Therefore, they may be used as a potent anticancer vaccine in addition to their broad antineoplastic cytotoxicity.

### Redirecting T cells to tumours

**Figure 6:**
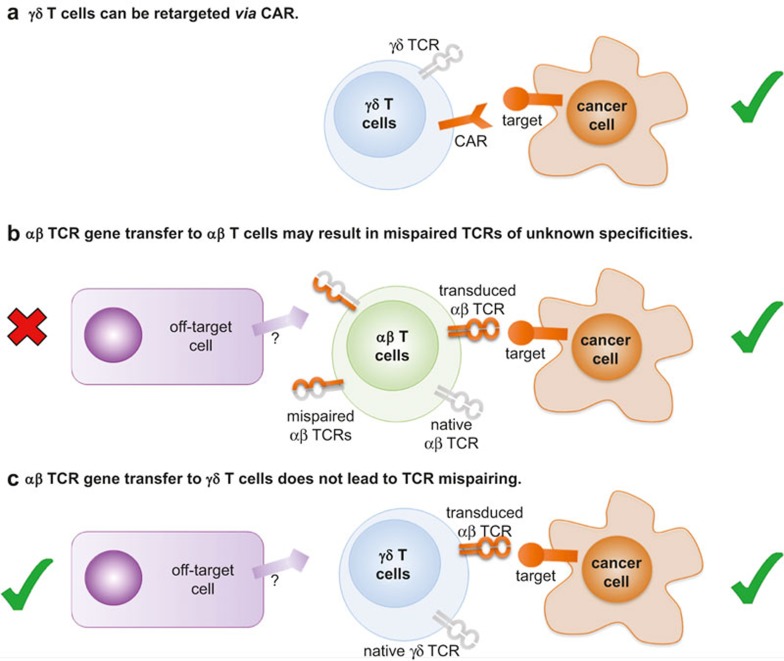
Genetic modification of γδ T cells for adoptive therapy approaches to cancer. (**a**) γδ T cells can be redirected to kill cancer cells using a chimeric antigen receptor (CAR) made from an antibody that targets a tumour-specific molecule at the cancer cell surface. (**b**) αβ T cells can be redirected to kill cancer cells by transducing them with a cancer-specific αβ TCR. Such αβ TCR gene transfer could result in the expression of up to four different αβ TCRs at the T cell surface: (i) the endogenous TCR; (ii) the transduced TCR; (iii) a hybrid TCR consisting of the endogenous TCRα chain paired with the transduced TCRβ chain; and (iv) endogenous TCRβ chain paired with the transduced TCRα chain. Neither hybrid will have undergone the rigours of thymic selection and therefore these TCRs have the potential of being autoreactive. (**c**) Transduction of γδ T cells with an αβ TCR provides a means of circumventing the potential mispairing problem seen in **b**.

In the absence of known ligands, γδ T cells can be redirected to tumours using antibodies. The efficacy of bispecific antibodies, where one part recognised a tumour surface marker (for example, EpCAM on liver tumours or HER2/neu on pancreatic cancer) while the other binding site targets CD3 or the Vγ9 chain of the TCR, has been demonstrated in pre-clinical models.^81,82^ An interesting approach was described by Zheng *et al.* where they cloned out the extracellular domains of a Vγ9Vδ2 TCR from ovarian cancer tumour-infiltrating lymphocytes (TILs) and conjugated them with Fc domain of human IgG1.^83^ This bispecific construct bound to a range of ovarian cancer cells, recognising an unknown ubiquitous antigen, and mediated the killing of the cells via antibody-dependent cellular cytotoxicity (ADCC). ADCC can be mediated by binding of CD16 (FcγRIII) to the Fc region of IgGs, constituting yet another means of target recognition by γδ T cells, in addition to TCR and NKG2D. CD16 can be upregulated on both Vδ2^pos^ and Vδ2^neg^ T cells, depending on circumstances, while binding of its target may trigger either cytotoxicity or other effector functions (e.g. IFNγ secretion).^84,85^ A similar approach involves transducing T cells with chimeric antigen receptors (CARs; Figure 6). CARs are usually derived from single chain Fv fragments of antibodies, thus enabling the CAR-transduced T cells to recognise conformational epitopes independently of their TCR (recently reviewed in Maus *et al.*^86^). To date, most CAR transduction experiments have been conducted on αβ T cells – nevertheless, γδ T cells are also an appealing target, due to their broad antitumour cytotoxicity and numerous effector functions. Recently, Deniger *et al.* have transduced polyclonal γδ T cells with a CD19-specific CAR, demonstrating their efficacy in killing CD19^+^ leukaemia lines.^87^ CAR-mediated signalling resulted in a similar cytokine secretion profile as TCR activation, and induced unbiased expansion of γδ T cell subsets. Notably, CAR technology has been combined with the generation of induced pluripotent stem cells (iPSC) from human peripheral blood T cells.^88^ Such cells exhibited a phenotype closest to γδ T cells of all lymphocyte lineages and exerted similar *in vivo* antitumour activity to CAR-transduced γδ T cells.

Finally, T cells can be redirected to tumours by introducing an exogenous TCR of known anticancer specificity into patient-derived peripheral lymphocytes prior to adoptive transfer of these cells. Most TCR gene studies have involved transduction of αβ TCRs into αβ T cells.^89^ However, this strategy comes with the inherent risk of αβ TCR mispairing between the endo- and exogenous TCR chains, resulting in receptors of unknown and potentially autoreactive specificities (Figure 6b). Such autoreactivity has been observed both in *ex vivo* human samples^90^ and in mouse models.^91^ γδ T cells offer an attractive way to circumvent this problem as tumour-reactive αβ TCRs can be introduced into these cells without the risk of mispairing (Figure 6c).^92,93^ Additionally, γδ T cells transduced with αβ TCRs retain the functionality of their original TCR and respond to stimuli transferred via either TCR with rapid, γδ-like kinetics.^94^ The main obstacle associated with αβ TCR transfer, however, is that γδ T cells usually do not express CD4 or CD8 co-receptors required for the efficient recognition of peptide-MHC. Thus efficient function might require co-transduction with a coreceptor^95^ or use of enhanced affinity TCRs.^96^ It is also possible to transduce peripheral lymphocytes (both of γδ and αβ origin) with a specific γδ TCR. Zhao *et al.* demonstrated that T cells transduced with a Vγ9Vδ2 TCR, modified with a CDR3δ loop specific for unidentified antigens on ovarian carcinoma, exerted an anticancer activity *in vivo*.^97^

Similarly, the transduction of a PAg-reactive Vγ9Vδ2 TCR into αβ T cells successfully redirected them towards cancer cells, as well as led to downregulation of their endogenous αβ TCRs, thus abrogating alloreactive responses.^98^ It is therefore likely that the detailed characterisation of cancer-specific γδ TCRs will bring about new studies examining the efficacy of such TCRs in the TCR gene transduction setting.

### Generating clinically relevant numbers of tumour-reactive γδ T cells

The main hurdle that needs to be overcome in order to use γδ T cells as a therapeutic is obtaining these cell in clinically meaningful numbers. For αβ T cell-based therapies, this is a relatively straightforward task as the requirements for effective αβ T cell expansion have been widely studied, and the cognate ligands can be readily used to stimulate the expanding cells.^99^ In the case of γδ T cells, the only known common ligands are PAgs which are recognised by the Vγ9Vδ2 subset. As aminobisphosphonates such as zoledronate cause accumulation of PAgs in the cells, they can be used to preferentially expand that T cell subset both *ex vivo*, prior to re-infusion, and directly in patients. However, PAgs and aminobisphosphonates expand only the Vγ9Vδ2 subset of γδ T cells, and this bias may have a negative impact on clinical efficacy. In an alternative approach, immobilised anti-γδ TCR antibody has been demonstrated to promote the expansion of all γδ T cell subsets, without modulating their antitumour function.^100,101^ This method, however, may lead to mitogen-induced T cell death and has not yet been used in a published clinical trial. Deniger *et al.* have recently proved that T cells with polyclonal γδ TCR repertoires can be expanded to large numbers (>10^9^) using IL-2, IL-21 and clinical grade artificial antigen-presenting cells, engineered to express co-stimulatory molecules CD19, CD64, CD86, CD137L and membrane-bound IL-15.^102^ Notably, the antigen-presenting cells were derived from a tumour line K562, broadly recognised by γδ T cells, and γ-irradiated prior to T cell exposure. The irradiation step could potentially enhance the recognition even further, as it leads to expression of cell stress molecules. Polyclonal γδ T cell lines showed superior toxicity compared to those expressing only Vδ2 chains, thus highlighting the possibility of enhancing γδ T cell-focused immunotherapy. Similarly, Anderson and colleagues used the same antigen-presenting cell line to generate unbiased γδ T cell products from peripheral blood of cancer patients.^84^

Finally, manipulations of the CD3 co-receptor can be applied to increase therapeutic efficacy of γδ T cells. Dopfer *et al.* recently showed that two anti-CD3 antibodies affect the functional activity of γδ T cells differentially.^103^ The reported results indicated that an anti-CD3 antibody, OKT3, induced strong proliferation and cytokine secretion with decreased cytotoxic potential, while another anti-CD3 antibody, UCHT1, enhanced tumour killing via Ras/Erk and PI3K/Akt pathways, without an increase in cytokine secretion or proliferation. These results might pave the way for new therapeutic approaches focused on manipulation for CD3-mediated signal transduction to favour expansion of large numbers of γδ T cells, with desired effector functions such as cytotoxicity.

### Lessons learned from clinical trials

Numerous attempts to use γδ T cells in cancer immunotherapy have been made over the past decade, with variable efficacy and a good overall safety profile.

Two recent trials investigated the anticancer effect of zoledronate combined with low dose IL-2. In renal cell carcinoma, the combined treatment resulted only in a minor expansion of Vδ2^pos^ cells which diminished after repeated treatment cycles without achieving the objective response in any of the enrolled patients.^104^ A similar study by Kunzmann and colleagues showed no objective response in patients with solid tumours (melanoma and renal cell carcinoma), and a partial response in acute myeloid leukaemia patients.^105^ Surprisingly, the treatment resulted in increased vascular endothelial growth factor levels, an indication of augmented angiogenesis, having a negative impact on the therapeutic outcome. No severe side effects (apart from grade 4 fever) were observed. A recent phase IV clinical trial of zoledronate in cancer-free patients demonstrated that the severity of transient inflammation-related side effects, caused by the treatment, could easily be predicted by *ex vivo* measurements of IFN-γ production by zoledronate-activated peripheral blood cells.^106^ Notably, repeated zoledronate treatment led to the depletion of the central memory subset of blood Vγ9Vδ2 T cells, as well as a long-lasting decrease of their absolute count.^106,107^ This phenomenon may be caused by peripheral blood neutrophils which have been shown to ingest zoledronate and subsequently inhibit the proliferation of the T cells by production of hydrogen peroxide.^108^ Repeated stimulation with PAgs, either accumulated in response to aminobisphosphonates or as a result of an accelerated mevalonate pathway, can also lead to a differentiation shift of Vγ9Vδ2 T cells and functional exhaustion.^109^ Zoledronate therefore appears to be insufficient to generate an effective antitumour response *per se* – nevertheless, two multicentre studies demonstrated that treatment with zoledronate combined with chemotherapy improved the survival of breast cancer and multiple myeloma patients.^110,111^

The adoptive cell transfer (ACT) of *ex vivo* expanded γδ T cells appears to be a more effective treatment option than using zoledronate as a single agent. In metastatic renal cell carcinoma, two groups reported re-infusing autologous PAg-expanded Vδ2^pos^ T cells.^112,113^ When the T cells were infused with IL-2 only, 6 out of 10 patients experienced stabilisation of the disease whereas the infusion of the cells with both IL-2 and zoledronate resulted in 5 out of 10 disease stabilisations and 1 complete response. However, as both studies included only a small number of patients, it is possible that the observed superior efficacy of using both IL-2 and zoledronate was purely coincidental. When cells were expanded using IL-2 and zoledronate but infused without further exogenous stimulation, the partial response was observed in 3 out of 10 patients with various solid tumours.^114^ As the patients were treated with chemotherapy together with ACT, the response could be contributed to the additive/synergistic effect of the combination therapy. A similar effect was observed by Nicol and colleagues as the complete response (1/17) and partial response (2/17) patients received both cell infusion and chemo/hormone therapy.^115^ However, the patient cohorts in these studies were small and highly diverse, in terms of cancer type, additional therapy and composition of the T cell infusion. These variables make it difficult to assign the exact clinical contribution of γδ T cells. ACT of γδ T cells and other cytotoxic cell types can also increase the efficacy of radiofrequency ablation.^116^ The aforementioned trials utilised patients' autologous peripheral blood T cells for ACT – however, in some instances the effector functions of autologous T cells are severely impaired.^109^ A recent study by Wilhelm *et al.* demonstrated that it is possible to infuse patients with a γδ T cell product obtained from haploidentical donors, with no evidence of graft versus host disease, showing substantial clinical efficacy (3 out of 4 patients with advanced haematological malignancies experienced complete regression).^117^ The donor γδ T cells persisted for 28 days and expanded *in vivo*, in response to exogenous IL-2 and zoledronate. Another group also showed that prolonged persistence of infused γδ T cells can occur without IL-2 supplementation, probably relying on endogenous IL-15.^118^ Moreover, exogenous IL-18 can be a potent co-stimulator of γδ T cell proliferation.^119,120^

Overall, while γδ T cell therapy has a good safety profile, clinical performance to date has been underwhelming.^121^ Anticipated discoveries of the various ligands recognised on tumour cells by the Vδ2^neg^ T cell subset will lead to a better understanding of how these cells operate and an increased capacity to beneficially harness these mechanisms for immunotherapy.

## CONCLUSIONS AND FURTHER DIRECTIONS

The evolutionary conservation of γδ T cells in all jawed vertebrates and of an analogous third lymphocyte lineage in their jawless ancestors^2^ attests to the critical importance of these cells in the maintenance of immune integrity. γδ T cells are known to play an important role in infection^16^; further recent discoveries have shown that this T cell lineage can respond to stress signals and thereby play an important role in cancer immunosurveillance. Indeed, mice lacking the γδ TCR have increased cancer risk.^17^ Despite the importance of γδ T cells, very few ligands for the human γδ TCR have been confirmed by biochemical and biophysical data and this field is still in its infancy. Interestingly, certain similarities exist between the few known human γδ TCR ligands that might point to some generalised mechanisms by which these cells patrol the periphery. BTN3A1, MIC A/B and CD1-family proteins belong to the immunoglobulin superfamily (while EPCR is highly homologous to CD1d), and both infections and cellular stress seem to play a role in activating γδ T cells *via* these immunoglobulin structures. Microbial infections lead to a stimulatory conformational change and/or direct metabolite presentation by BTN3A1 while herpesvirus (CMV, EBV) infections cause upregulation of EPCR, MIC A and hMSH2, respectively. Similarly, cell stress, including neoplasms, can involve deregulation of the mevalonate pathway^122^ and subsequent PAg activation of BNT3A1 in addition to ectopic expression of cell stress markers such as EPCR, MIC and hMSH2, while CD1-family proteins can present endogenous lipids acting as a marker of malignant transformation (CD1c)^42^ or viral infection (CD1d).^123^ Interestingly, both CD1d and EPCR are capable of lipid presentation, and both can be recognised by γδ T cell clones regardless of the lipid cargo – thus indicating that both molecules can act as an immunogenic marker *per se*.^35,47^ Requirement for a wider cell stress context for γδ T cell recognition potentially restrains these immune cells and provides a failsafe against autoimmune reactions. Cancer-associated γδ TCR targets are essentially self-antigens. Accordingly, γδ TCR interactions with the known targets are of low affinity (K_D_ = 10^–4^–10^–6^ M; Tables 1–3), similar to αβ TCR affinities for self-derived peptides.^124,125^ To date, only two structures of human γδ TCR in complex with a ligand have been resolved (Figure 4). In both cases (CD1d-sulphatide and CD1d-αGalCer), the germline-encoded CDR loops engaged the antigen-presenting CD1d molecule while hypervariable CDR3 loops made contacts with the lipid ligand, suggesting that discrimination between subtly different lipid cargoes is possible. In contrast, PAg recognition may be mediated mostly by germline-encoded CDR loops from Vδ2 and/or Vγ9 chains as PAg-BTN3A1 can be recognised by a wide range of different Vδ2 Vγ9 T cell clones. However, the exact mode of PAg recognition awaits a BTN3A1: γδ TCR complex structure.

In summary, γδ T cell therapy for cancer has demonstrated a good overall safety profile although results to date have not lived up to their early promise. Nevertheless, we anticipate that many more ligands will be found for these important immune cells in the next 5 years thereby greatly expanding the therapeutic horizon. Therapeutic approaches to cancer treatment based around the αβ TCR in gene transfer^99^ or as soluble bispecifics^126,127^ have burgeoned in the last two years. Importantly, γδ TCRs do not appear to suffer the limitation of being HLA-restricted so might offer exciting possibilities for new off-the-shelf, pan-population cancer immunotherapies in the very near future.

## Figures and Tables

**Table 1:** The interaction affinities of IPP binding to the intracellular or extracellular domains of BTN3A1, and of TCR binding to BNT3A1 in presence of IPP.

Interaction	K_D_ (μM)	Ref.
IPP: extracellular BTN3A1	69.9	31
IPP:B30.20	≈500	29
Vγ9Vδ2 TCR:BTN3A1(IPP)	340	31

**Table 2:** The interaction affinities of TCR binding the CD1d-lipid complex.

TCR	Ligand	K_D_ (μM)	PDB code	Ref.
Vγ4 Vδ1	CD1d-sulphatide	5.6	4MNG	36
Vγ5 Vδ1	CD1d-αGalCer	16	4LHU	37
Vδ1/Cα Vβ2	CD1d-αGalCer	0.066	4WO4	38

**Table 3:** γδ TCR and NKG2D binding affinities to stress-related ligands EPCR, hMSH2 and MIC A.

Receptor	Ligand	K_D_ (μM)	Ref.
Vγ4 Vδ5 TCR (LES)	EPCR	96	47
Vδ2 TCRs	hMSH2	N/A	54
NKG2D	hMSH2	0.132	54
Vγ4 Vδ1 TCR	MIC A	110–900	70
NKG2D	MIC A	0.3–21	70
